# Laccase-Catalyzed Decolorization of Malachite Green: Performance Optimization and Degradation Mechanism

**DOI:** 10.1371/journal.pone.0127714

**Published:** 2015-05-28

**Authors:** Jie Yang, Xiaodan Yang, Yonghui Lin, Tzi Bun Ng, Juan Lin, Xiuyun Ye

**Affiliations:** 1 College of Biological Sciences and Technology, Fuzhou University, Fuzhou, Fujian, China; 2 Fujian Key Laboratory of Marine Enzyme Engineering, Fuzhou, Fujian, China; 3 Technical Center, Fujian Entry-Exit Inspection and Quarantine Bureau, Fuzhou, Fujian, China; 4 School of Biomedical Sciences, Faculty of Medicine, The Chinese University of Hong Kong, Shatin, New Territories, Hong Kong, China; University Of Helsinki, FINLAND

## Abstract

Malachite green (MG) was decolorized by laccase (LacA) of white-rot fungus *Cerrena* sp. with strong decolorizing ability. Decolorization conditions were optimized with response surface methodology. A highly significant quadratic model was developed to investigate MG decolorization with LacA, and the maximum MG decolorization ratio of 91.6% was predicted under the conditions of 2.8 U mL^-1^ LacA, 109.9 mg L^-1^ MG and decolorization for 172.4 min. Kinetic studies revealed the *K*m and *k*cat values of LacA toward MG were 781.9 mM and 9.5 s^-1^, respectively. UV–visible spectra confirmed degradation of MG, and the degradation mechanism was explored with liquid chromatography–mass spectrometry (LC-MS) analysis. Based on the LC-MS spectra of degradation products, LacA catalyzed MG degradation via two simultaneous pathways. In addition, the phytotoxicity of MG, in terms of inhibition on seed germination and seedling root elongation of *Nicotiana tabacum* and *Lactuca sativa*, was reduced after laccase treatment. These results suggest that laccase of *Cerrena* was effective in decolorizing MG and promising in bioremediation of wastewater in food and aquaculture industries.

## Introduction

Malachite green (MG) is a triphenylmethane dye used in aquaculture to control protozoan and fungal infections of farmed fish. MG is also used in food, medical and textile industries. MG is readily absorbed by fish and reduced to leucomalachite green (LMG), a colorless, toxic metabolite [1–3]. MG is environmentally persistent; MG and LMG can accumulate in fish tissues. MG has a wide toxicity spectrum covering microorganisms and higher eukaryotes [1,4]. Its toxic effects include organ damages, impaired growth and reproduction, developmental abnormalities and mutagenic/carcinogenic potentials [3,5]. Furthermore, the dye effluent can be disposed of untreated into water bodies and be used for agriculture, thereby affecting soil fertility [6]. Although banned in many countries, MG is still used in many areas worldwide due to its efficacy, low cost and availability and thereby continues to pose threats to the environment, food safety and human health. Physical and chemical methods of MG removal have been proposed, such as adsorption [7] and photodegradation [8,9]. These processes are often ineffective, not economically feasible and can cause secondary pollution. On the other hand, biodegradation has shown promise in MG treatment [10,11]. Triphenylmethane reductase and cytochrome P450 mediate MG reduction to LMG [5,12–14]. Therefore, enzyme-catalyzed MG oxidation without forming LMG derivatives is desired for MG decolorization and detoxification [2,4,15–21].

White rot fungi are effective lignin degraders by secreting ligninolytic enzymes including lignin peroxidases (EC 1.11.1.14), manganese peroxidases (EC 1.11.1.13) and laccases (EC 1.10.3.2) [22]. All these enzymes can degrade MG, and the specific enzyme in action depends on the fungus [21]. Laccases have attracted much research interest because they are environmental friendly and can oxidize a wide variety of phenolic and non-phenolic compounds. Belonging to the family of multicopper enzymes, laccases contain four (one type 1, one type 2 and two type 3) copper atoms per molecule and catalyze one-electron oxidation of substrates concomitant with four-electron reduction of molecular oxygen to water [22,23]. Laccase have important applications in various processes such as dye decolorization and detoxification, wastewater treatment and bioremediation [24]. The substrate range of laccases can be further expanded with small molecular weight mediators, such as 2,2’-azino-bis (3-ethylbenzothiazoline-6-sulfonate) (ABTS) and 1-hydroxybenzotriazole (HBT). The laccase/mediator system can facilitate the oxidation pathway; different radicals may be formed, depending on whether a mediator is involved or not [22,25–28].

In search of efficient laccase producers to promote industrial applications of laccases, we isolated a novel white rot fungus *Cerrena* sp. strain HYB07 with high laccase yield and strong decolorization ability towards structurally different dyes, such as MG [29]. In the present work, a major laccase produced by HYB07, namely LacA, was used to decolorize MG in the absence of a mediator. Response surface methodology (RSM) was adopted to optimize decolorization conditions with respect to dye concentration, enzyme activity and reaction time. The degradation products were identified with liquid chromatography–mass spectrometry (LC-MS), and a novel degradation model of MG was proposed.

## Materials and Methods

### Chemicals and organism

MG and ABTS were purchased from Sigma-Aldrich. All reagents were of analytical grade. The white rot fungus *Cerrena* sp. strain HYB07 was maintained on potato dextrose agar slants at 4°C and stored in the culture collection of College of Biological Sciences and Technology, Fuzhou University, China.

### Enzyme production and purification

Fermentation of *Cerrena* sp. H’YB07 was carried out in potato dextrose broth supplemented with 0.5% yeast extract and 0.4 mM CuSO_4_. After 3 d, the fermentation broth was harvested, diluted in 25 mM Tris-HCl buffer (pH 7.5) and applied to a HiTrap DEAE FF column (GE Healthcare). Adsorbed proteins were eluted with a linear gradient of 0–1 M NaCl in 25 mM Tris-HCl buffer (pH 7.5). Fractions with laccase activity were collected and checked by SDS-PAGE. The major laccase produced by HYB07, LacA (GenBank accession number KF317949) [29], was purified to electrophoretic homogeneity. The specific activity of LacA was 1952.4 U mg^-1^. The enzyme activity assay was carried out in a citrate-phosphate buffer (50 mM, pH 3.0) at 45°C, and oxidation of ABTS was monitored spectrophotometrically at 420 nm (ε = 36,000 M^-1^ cm^-1^) for 5 min [29]. One unit of enzyme activity was defined as the amount of laccase required to oxidize 1 μmol ABTS per min.

### MG decolorization

The absorbance peak of MG was determined to be 614 nm by UV-visible analysis. Purified LacA was used to decolorize MG at 28°C in an incubator (ZXSD-1270, LABWIT Scientific, Shanghai, China) in the dark. The 4 mL reaction mixture contained 50 mM citrate-phosphate buffer (pH 6.0), dye and laccase. Decolorization efficiency was monitored at 614 nm with a UV-Vis spectrophotometer (U-2910, Hitachi, Japan) and calculated according to the following formula:

$$\text{D}\;\left(\%\right)=\left({\text{A}}_{0}-{\text{A}}_{1}\right)\;/\;{\text{A}}_{0}\;\text{x}\;100$$

Where D is the decolorization efficiency (%), A_0_ is MG absorption before LacA treatment, and A_1_ is the residual MG absorption after LacA treatment.

### Performance optimization of MG decolorization by RSM

Central composite design (CCD) was chosen for the optimization of MG decolorization process by LacA. Three independent variables, namely LacA concentration (*X*_1_), dye concentration (*X*_2_) and time (*X*_3_) were evaluated at five levels (Table 1), and the percentage of MG decolorization was the dependent variable (response). The following equation was used to establish the quadratic model:

$$Y=\beta_{0}+\beta_{1}X_{1}+\beta_{2}X_{2}+\beta_{3}X_{3}+\beta_{1}\beta_{2}X_{1}X_{2}+\beta_{1}\beta_{3}X_{1}X_{3}+\beta_{2}\beta_{3}X_{2}X_{3}+\beta_{11}X_{1}{}^{2}+\beta_{22}X_{2}{}^{2}+\beta_{33}X_{3}{}^{2}$$

where *Y* is the predicted response; *X*_1_, *X*_2_ and *X*_3_ are the coded factors; *β*_0_ is a constant coefficient; *β*_1_, *β*_2_, *β*_3_ are the linear coefficients; *β*_11_, *β*_22_, *β*_33_ are the quadratic coefficients; *β*_1_ *β*_2_, *β*_1_ *β*_3_, *β*_2_ *β*_3_ are the interactions of the coefficients.

**Table 1:** The CCD design and the observed responses for MG decolorization.

S. No.	LacA (U mL^-1^)	Dye (mg L^-1^)	Time (min)	Decolorization
	*X*_1_	*X*_2_	*X*_3_	(%)
1	-1 (1.0)	-1 (50.0)	-1 (60.0)	41.01
2	1 (3.0)	-1 (50.0)	-1 (60.0)	58.05
3	-1 (1.0)	1 (150.0)	-1 (60.0)	33.18
4	1 (3.0)	1 (150.0)	-1 (60.0)	61.56
5	-1 (1.0)	-1 (50.0)	1 (180.0)	69.70
6	1 (3.0)	-1 (50.0)	1 (180.0)	91.20
7	-1 (1.0)	1 (150.0)	1 (180.0)	73.61
8	1 (3.0)	1 (150.0)	1 (180.0)	89.24
9	-1.68 (0.32)	0 (100.0)	0 (120.0)	31.33
10	1.68 (3.68)	0 (100.0)	0 (120.0)	78.97
11	0 (2.0)	-1.68 (15.91)	0 (120.0)	74.10
12	0 (2.0)	1.68 (184.09)	0 (120.0)	70.00
13	0 (2.0)	0 (100.0)	-1.68 (19.09)	19.58
14	0 (2.0)	0 (100.0)	1.68 (220.91)	79.42
15	0 (2.0)	0 (100.0)	0 (120.0)	84.49
16	0 (2.0)	0 (100.0)	0 (120.0)	81.24
17	0 (2.0)	0 (100.0)	0 (120.0)	79.07
18	0 (2.0)	0 (100.0)	0 (120.0)	74.03
19	0 (2.0)	0 (100.0)	0 (120.0)	77.38
20	0 (2.0)	0 (100.0)	0 (120.0)	83.83

Design-Expert version 8.0 (Stat-Ease Inc., Minneapolis, USA) was used for experimental design and statistical analysis. Validation of the optimum decolorization results predicted by the model was conducted in triplicate.

### Kinetic study

Substrate specificity of LacA for MG was determined with nonlinear regression of the Michaelis-Menten equation by using GraphPad Prism version 5.0 (GraphPad Software, Inc., USA). The 4 mL reaction consisted of MG (20, 50, 80, 100, 200, 300, 500, 1000 and 2000 mg L^-1^), LacA (3 U mL^-1^) in 50 mM citrate-phosphate buffer (pH 6.0). All experiments were performed in triplicate.

### Identification of degradation products

For LC-MS, MG decolorization was performed at 25°C in water instead of buffer. Aliquots (2 μL) were injected into an HPLC system (Agilent 1200 Series, equipped with a Phenomenex Luna C-18 analytical column of 2.0 mm x 150 mm length and 3 μm particle size) coupled with Agilent 6224 Accurate-Mass Time of Flight (TOF) MS. The compounds were resolved by using solvent A: 5 mM ammonium acetate supplemented with 0.5% formic acid and solvent B: acetonitrile. The flow rate was kept at 0.2 mL min^-1^. A linear gradient was set as follows: t = 0–2, A = 95; t = 4–5, A = 40; t = 7–11, A = 10; t = 12–15, A = 95. The column effluent was introduced into the electrospray ionization source of the mass spectrometer in positive ion mode. The MS parameters were as follows: capillary voltage 3.5 kV; nebulizer pressure 50 psi; drying gas flow 11 L min^-1^; drying gas temperature 360°C; fragmentor voltage 130 V. LC-TOF MS accurate mass spectra were recorded across the range 70–400 *m*/*z*. Data processing was carried out with Applied Biosystems/MDS-SCIEX Analyst QS software (Frankfurt, Germany) with accurate mass application-specific additions from Agilent MSD TOF software. Accurate-mass internal mass calibration was performed automatically using a dual-nebulizer ion source. The reference masses were 121.0509 *m*/*z* and 922.0098 *m*/*z*.

### Phytotoxicity study

Toxicity of MG before and after LacA decolorization was assayed with *Nicotiana tabacum* and *Lactuca sativa* with respect to seed germination and seedling root (radical) elongation. The seeds were placed in petri dishes (at least 30 seeds each plate) on filter paper pre-wet with MG solution (100 mg L^-1^) before and after LacA treatment. The petri dishes were sealed with parafilm and stratified at 4°C in the dark for 3 d to promote synchronous seed germination. The plates were then transferred to a plant growth chamber (PRX-250B, Saife Instruments, Ningbo, China) at 22°C. Distilled water was used as the control. Germination rate and root length were determined after 7 d. Radical emergence was used to determine germination; radical growth was measured by using the Image-Pro software (Media Cybernetics, San Diego, CA, USA).

## Results and Discussion

### Performance optimization of MG decolorization by LacA

Since the enzyme-mediated decolorization process is influenced by parameters such as dye concentration, enzyme activity and decolorization time, optimization of LacA-mediated MG decolorization was carried out. RSM is a statistical technique for optimization of multiple variables to achieve the best performance conditions with fewest possible experiments. CCD was chosen to determine the optimum requirement of enzyme (*X*_1_), dye (*X*_2_), and time (*X*_3_) for maximum dye decolorization (Table 1). The mathematical expression of the relationship to MG decolorization with the variables *X*_1_, *X*_2_ and *X*_3_ is as follows:

$$Y\;\left(\%\;\text{decolorization}\right)=79.78+11.91\;X_{1}-0.68\;X_{2}+16.88\;X_{3}+0.68\;X_{1}\;X_{2}-1.03\;X_{1}\;X_{3}+0.78\;X_{2}\;X_{3}-7.30\;X_{1}{}^{2}-1.32\;X_{2}{}^{2}-9.29\;X_{3}{}^{2}$$

**Fig 1:**
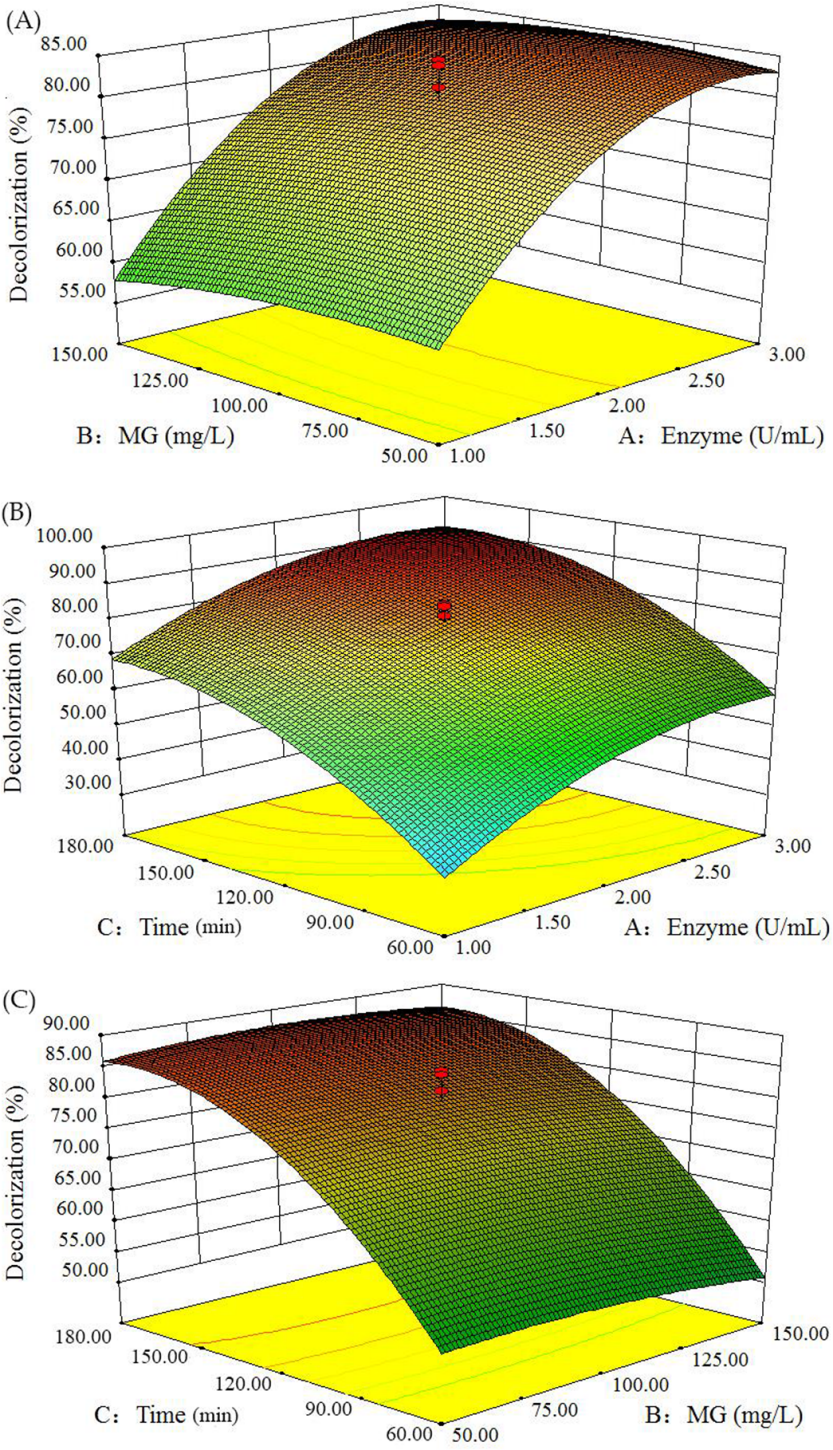
Response surface plots of the interaction between different variables. (A) Concentrations of enzyme and MG. (B) Concentration of enzyme and time. (C) Concentration of MG and time.

ANOVA of the regression model demonstrated a high significance (*P* < 0.0001) of the model and an insignificant lack of fit (Table 2). The determination coefficient R^2^ was 0.9581, and adjusted R^2^ (Adj-R^2^) was 0.9204. Adequate precision, a measure of the signal to noise ratio, was 15.93, indicating an adequate signal. Variables *X*_1_, *X*_3_, *X*_1_^2^ and *X*_3_^2^ were significant model terms. Interactions between the studied variables for dye decolorization are shown in 3D surface plots (Fig 1).

**Table 2:** ANOVA for the response surface quadratic model.

Source	Sum of squares	*df*	Mean square	*F* value	Prob > *F*
Model	7689.83	9	854.43	25.40	< 0.0001*
*X*_1_	1937.76	1	1937.76	57.61	< 0.0001*
*X*_2_	6.30	1	6.30	0.19	0.6745
*X*_3_	3892.42	1	3892.42	115.72	< 0.0001*
*X*_1_ *X*_2_	3.74	1	3.74	0.11	0.7457
*X*_1_ *X*_3_	8.54	1	8.54	0.25	0.6252
*X*_2_ *X*_3_	4.92	1	4.92	0.15	0.7101
*X*_1_^2^	767.02	1	767.02	22.80	0.0008*
*X*_2_^2^	25.16	1	25.16	0.75	0.4074
*X*_3_^2^	1244.60	1	1244.60	37.00	0.0001*
Residual	336.35	10	33.64		
Lack of Fit	256.63	5	51.33	3.22	0.1126
Pure Error	79.72	5	15.94		
Cor Total	8026.18	19			

*Significant (*P* < 0.05).

The optimum levels of coded variables for maximum decolorization (91.64%) were predicted as *X*_1_ = 0.763, *X*_2_ = 0.199 and *X*_3_ = 0.874. These values correspond to LacA = 2.76 U mL^-1^, MG = 109.93 mg L^-1^ and time = 172.44 min. Experimental validation (LacA = 2.8 U mL^-1^, MG = 110 mg L^-1^ and time = 172 min) was carried out using the optimized variables identified by RSM, and a decolorization efficiency of 89.88% with a 3.23% experimental error was obtained, which was close to the predicted value of 91.64%. The good correlation between the predicted and actual optimized decolorization efficiencies verified the validity of the response model.

CCD has been successfully applied for modeling and optimization of biological decolorization processes of dyes including reactive black 5 [30,31], Remazol Brilliant Blue R [32] and indigo carmine [30]. In these CCD studies, HBT is the most frequently used laccase mediator in order to achieve high decolorization efficiencies. For example, a maximum decolorization efficiency of approximately 96% was predicted with the conditions of 95.80 mg L^-1^ MG, 2.16 U mL^-1^ partially purified laccase from *Pleurotus florida*, 0.85 mM HBT and 3.02 h at pH 6.0 and 37 °C [20]. Despite the efficacy of laccase mediators in enhancing dye decolorization, they add processing costs and can have adverse impacts on the enzyme activity or the environment [15,16,28]. In our hands, at the same pH value of 6.0 as previous work [20], a similarly high decolorization efficiency was attained in the absence of a mediator at 28°C, demonstrating the potential and benefits of LacA in industrial applications. LacA was also efficient and eco-friendly compared with a recombinant manganese peroxidase isozyme H4, the latter decolorized 100 mg L^-1^ MG to a degree of 72% with MnSO_4_ and hydrogen peroxide [33].

### Kinetic study

The kinetic parameters of LacA-mediated MG decolorization were estimated from nonlinear regression of the Michaelis-Menten equation. The *K*_m_ and *k*_cat_ values were 781.9 mM and 9.5 s^-1^, respectively. Therefore, LacA had lower affinity as well as turnover rate for MG than for ABTS (93.4 μM and 2468.0 s^-1^) [29]. The *K*_m_ value (781.9 mM) was also greater than that of a triphenylmethane reductase from *Citrobacter* sp. Strain KCTC 18061P (0.53 mM); the reductase converts MG to LMG [12].

### LC-MS analysis of MG degradation products

**Fig 2:**
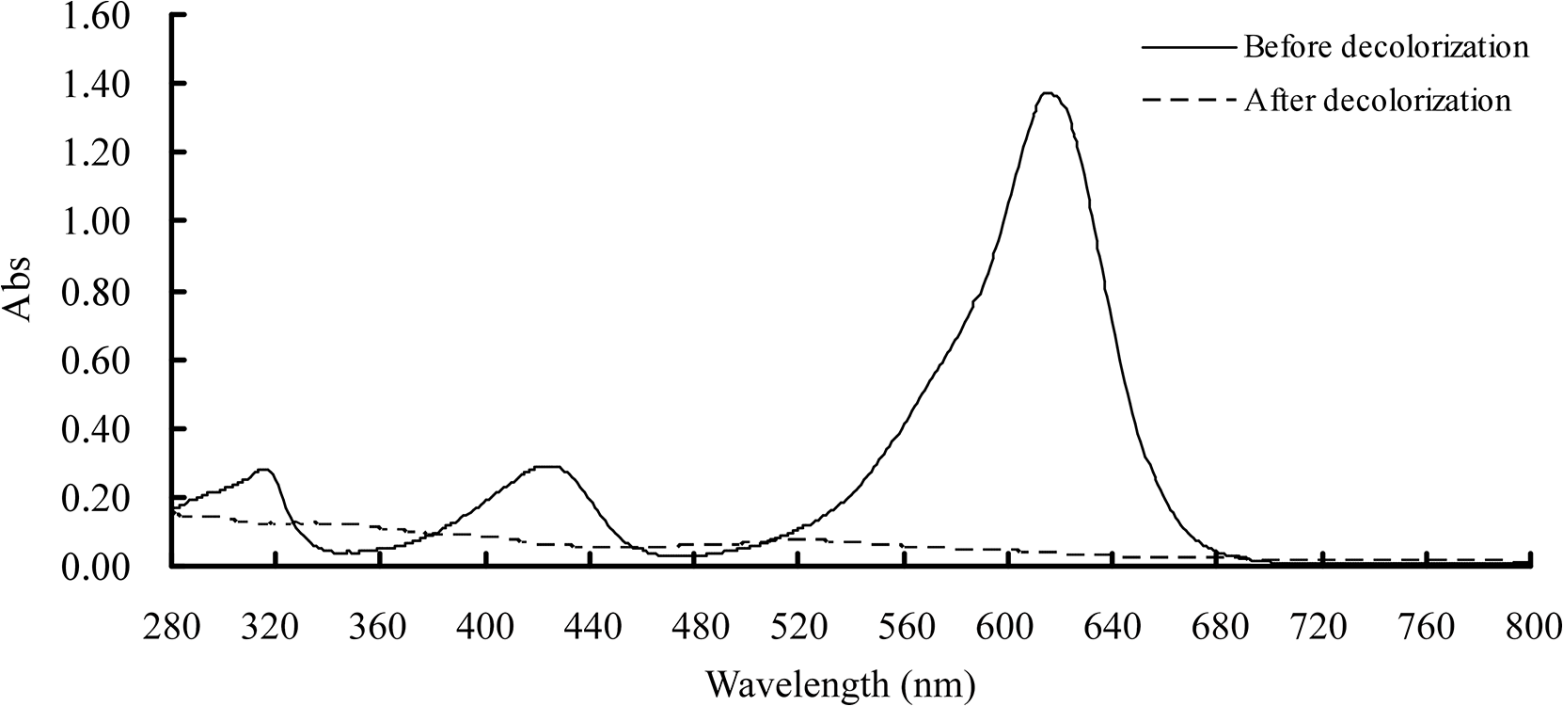
UV-visible spectra of MG before and after treatment with LacA.

**Fig 3:**
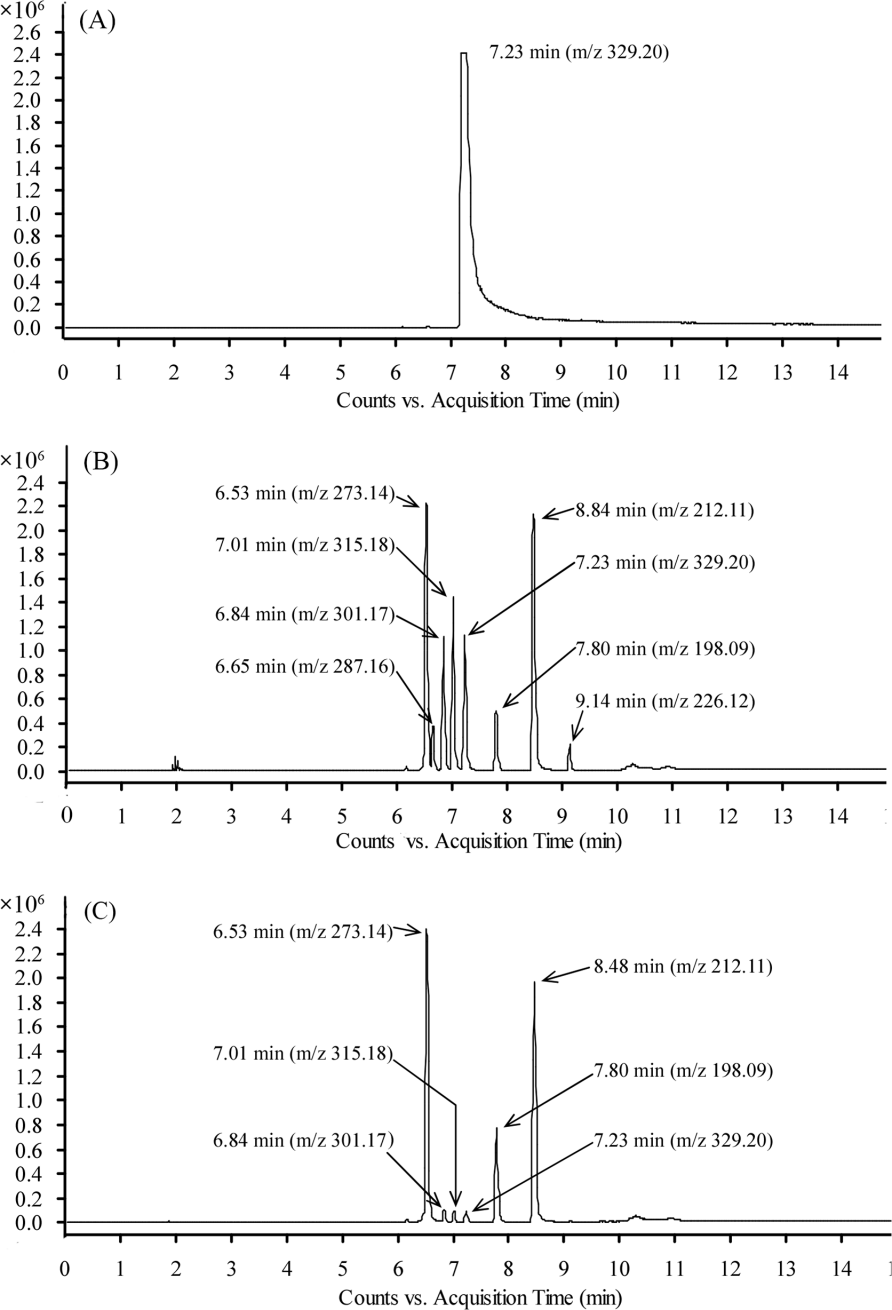
LC-TOF MS analysis of MG degraded by LacA. (A) MG control. (B) MG degradation after 60 min. (C) MG degradation after 180 min.

UV-visible spectra of the MG solution showed not only disappearance of the major peak of MG absorbance at 614 nm, but also two minor peaks at 315 nm and 425 nm, respectively, after LacA treatment (Fig 2), implying that decolorization was a result of biodegradation. This was confirmed by LC-TOF MS analysis. MG (*m*/*z* 329.20) concentration significantly decreased with LacA decolorization (Fig 3). Furthermore, unlike MG transformation with fungi *Cunninghamella elegans* [5], *Penicillium pinophilum* and *Myrothecium roridum* [13] or bacteria *Exiguobacterium* sp. [14] and *Citrobacter* sp. [12], in LacA-mediated MG degradation, reduction of MG to LMG was not detected.

**Fig 4:**
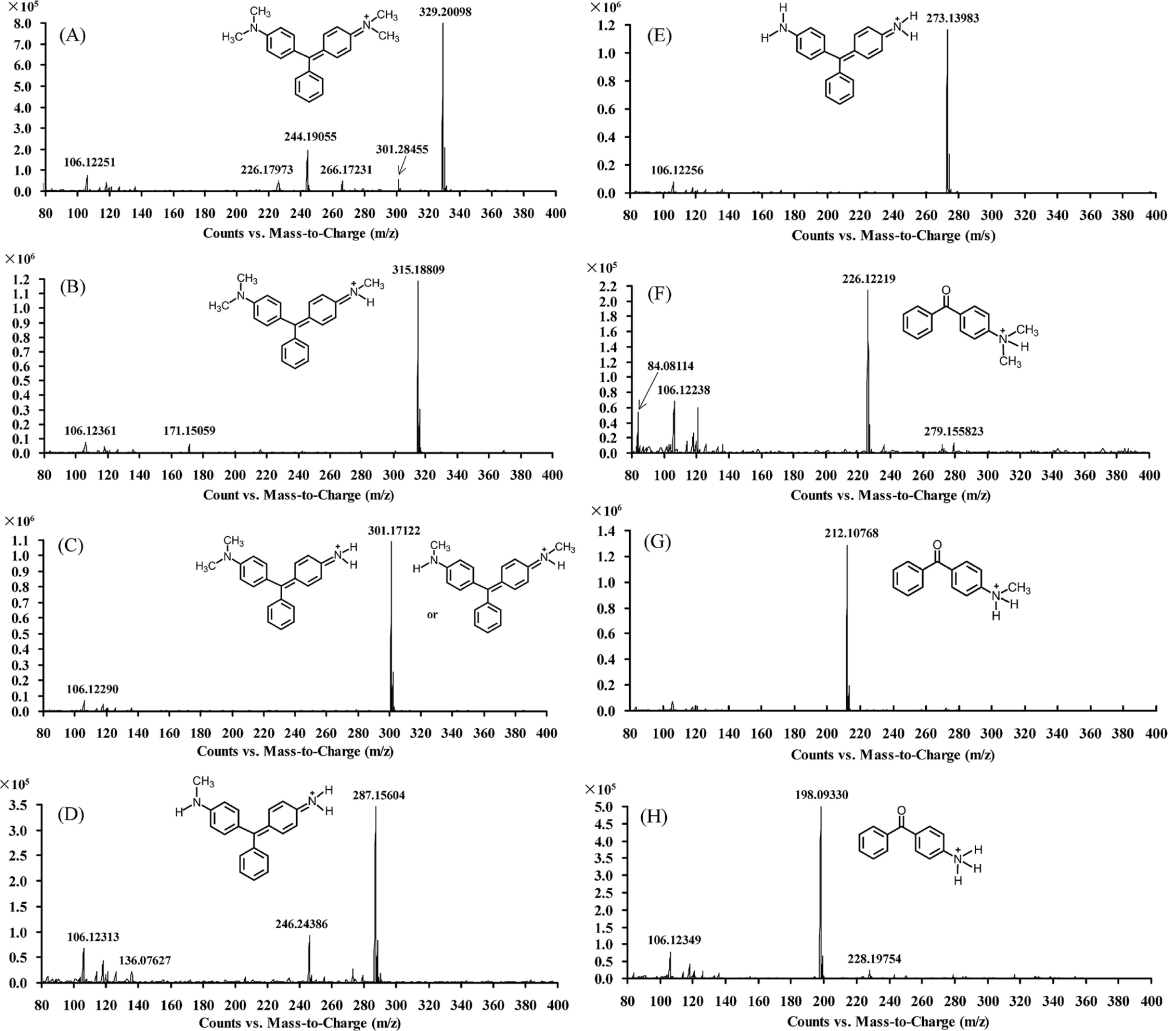
The mass spectra of MG and its degradation products. (A) MG, *m*/*z* 329.20 (retention time 7.23 min). (B) desmethyl MG, *m*/*z* 315.18 (retention time 7.01 min). (C) didesmethyl MG, *m*/*z* 301.17 (retention time 6.84 min). (D) tridesmethyl MG, *m*/*z* 287.16 (retention time 6.65 min). (E) tetradesmethyl MG *m*/*z* 273.14 (retention time 6.53 min). (F) (dimethyl amino-phenyl)-phenyl-methanone, *m*/*z* 226.12 (retention time 9.14 min). (G) (methyl amino-phenyl)-phenyl-methanone, *m*/*z* 212.11 (retention time 8.48 min). (H) (amino phenyl)-phenyl methanone, *m*/*z* 198.09 (retention time 7.80 min).

Development of a feasible decolorization scheme requires identification and evaluation of toxicity/mutagenicity of the major and stable products of the process [10,11,18]. Here, LC-TOF MS identified seven intermediates of LacA-catalyzed MG transformation (Fig 4). Among these intermediates, tetradesmethyl MG (*m*/*z* 273.14), (methyl aminophenyl)-phenyl-methanone (*m*/*z* 212.11) and (amino phenyl)-phenyl methanone (*m*/*z* 198.09) were more persistent products but diminished after prolonged incubation (e.g., overnight incubation).

### LacA-mediated MG degradation

**Fig 5:**
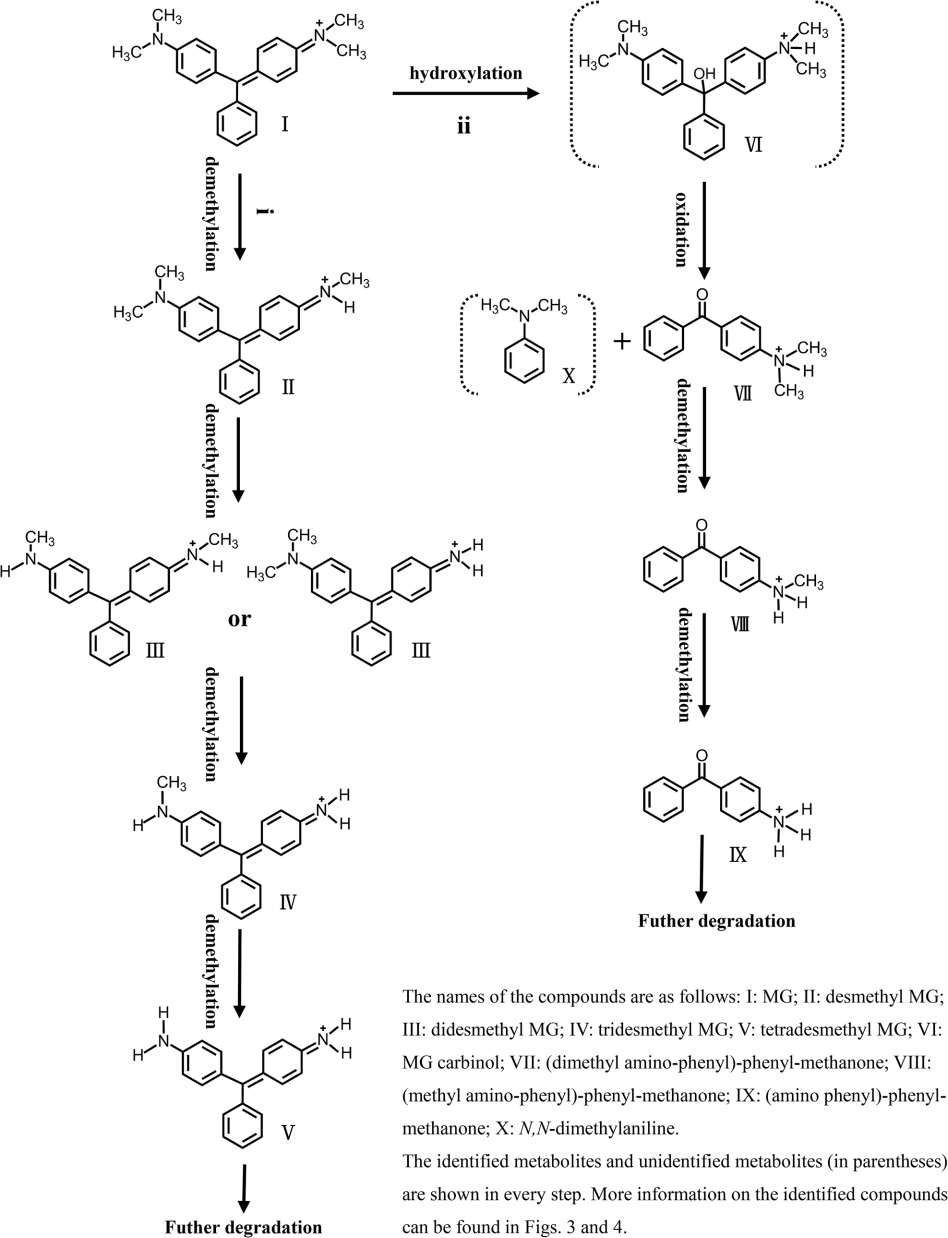
Proposed mechanism of LacA-mediated MG degradation. i, pathway I; ii, pathway II.

Based on the intermediates identified as well as previous studies on MG degradation, we proposed a model for LacA-mediated MG degradation consisting of two parallel and competing degradation pathways (Fig 5). Pathway I started with successive *N*-demethylation of MG, similar to proposed pathways of MG breakdown catalyzed by a laccase [16,18]. It is noteworthy that *N*-demethylation (i.e., complete conversion of MG to desmethyl MG, didesmethyl MG and tridesmethyl MG) does not result in MG decolorization. Further degradation or polymerization must occur to destroy the chromophore [16].

Besides pathway I, LacA also mediated MG degradation via a second pathway (pathway II). In this pathway, MG was first hydroxylated to its carbinol form [34], which was quickly broken down at the bond between the central carbon atom and the *N*,*N*-dimethylamino phenyl ring into (dimethyl amino-phenyl)-phenyl-methanone and *N*,*N*-dimethylaniline. (dimethyl amino-phenyl)-phenyl-methanone was then sequentially *N*-demethylated to (amino phenyl)-phenyl methanone. This pathway has not been previously reported for MG transformation catalyzed by only laccase (without a mediator), although it is the pathway of choice in the presence of a laccase mediator [18].

Simultaneous presence of both pathways in our model probably contributed to fast and efficient degradation of MG and could be accounted for by the speculated high redox potential of LacA. Bioinformatics analysis suggests that LacA is a high-redox-potential laccase, considering that three amino acids, namely Ser113, Glu456 and Phe459, conserved among fungal laccases with high redox potentials, were also present in LacA [23,35].

Reported fungal laccases have diverse redox potentials ranging from 400 to 800 mV, which are normally higher than those of plant laccases and other blue copper oxidases [22]. The efficiency of substrate oxidation by a laccase depends on the difference between the redox potentials of the substrate and the type 1 Cu. Oxidation of substrates with redox potentials higher than those of laccases, such as non-phenolic compounds, can be facilitated by electron transfer mediators. A laccase mediator oxidized first by a laccase can in turn oxidize non-phenolic substrates, thus enhancing the performance and efficiency of the laccase. For example, after oxidation by a laccase, di-cation ABTS^2+^ has a redox potential of 885 mV and oxidizes the substrate via an electron transfer route. In contrast, another synthetic mediator HBT or natural phenolic mediators follow a hydrogen atom transfer mechanism [22]. Laccase/mediator oxidation of the substrate may proceed differently from laccase oxidation of the substrate without a mediator [22,26–28]. Indeed, based on absorbance maximum changes, Papinutti and Forchiassin proposed that the mechanism of MG modification by *Fomes sclerodermeus* laccase is different when HBT is added [25]. Later, Chhabra et al. identified two independent, mutually exclusive degradation pathways of MG by laccase in the presence and absence of a mediator. With the laccase/ABTS system, MG is hydroxylated and broken down (pathway II), while laccase alone initiates MG transformation with stepwise *N*-demethylation (pathway I) [18]. This was not the case in our analysis; MG degradation with LacA or LacA/ABTS rendered identical intermediates from both pathways.

### Phytotoxicity study

Toxicity of MG solution before and after LacA treatment was evaluated with *Nicotiana tabacum* and *Lactuca sativa* in terms of seed germination and seedling root elongation (Table 3). While complete germination was observed in the water control, MG inhibited germination of both *Nicotiana tabacum* and *Lactuca sativa* seeds by 36% and 75%, respectively. Decreased seedling root elongation by MG was also observed, and the average seedling root length in MG was approximately only 30% of that in the water control. After LacA-mediated degradation, inhibition of MG on seed germination was eliminated, and MG inhibition on root elongation was alleviated. Incomplete restoration of radical length indicated that compared to germination, seedling root elongation was more sensitive to residual MG metabolites present after LacA-catalyzed decolorization. Similar to our phytotoxicity test results, MG impaired seed germination and root elongation rates of *Phaseolus mungo* and *Triticum aestivum*, and MG degradation by laccase from bacteria *Kocuria rosea* [36] or white-rot fungus *Pleurotus ostreatus* [19] resulted in products with lower toxicity on plant growth. Du et al. have reported complete and partial elimination of germination inhibition for Lucerne (*Medicago sativa* Linn.) and Chinese cabbage (*Brassica chinensis* Linn.) seeds, respectively, after MG removal with *Pseudomonas* sp. strain DY1 for 12 h [37]. Seedling root length of both plant species in treated MG solution was also partially restored to the level of the water control [37]. Reduction of phytotoxicity of other dyes, such as RBBR [32] and Red BLI [6], upon enzymatic decolorization has also been described, and incomplete reversion of root elongation in treated dye solutions are often observed.

**Table 3:** Phytotoxicity study of MG and the degradation products.

	*Nicotiana tabacum*			*Lactuca sativa*		
	Water	Untreated MG solution	Treated MG solution	Water	Untreated MG solution	Treated MG solution
Germination rate (%)	100	64	100	100	25	97
Root length (mm)	10.55 ± 0.21	3.52 ± 0.18	7.74 ± 0.19	22.53 ± 0.33	6.33 ± 0.24	16.87 ± 0.36

The data are means ± SE.

## Conclusions

MG decolorization by a novel laccase, LacA, was optimized with CCD to achieve a maximum decolorization efficiency of 91.64% in the absence of a laccase mediator. Based on the intermediates identified by LC-MS, LacA catalyzed MG transformation via two separate, co-existing pathways, likely owing to the high redox potential of the enzyme and contributing to effective decolorization and degradation of MG. Accompanying decolorization, LacA reduced toxicity of MG. LacA is a promising candidate for applications in MG removal as well as bioremediation of food and aquaculture wastewater.

## References

[1] Bergwerff AA, Scherpenisse P. Determination of residues of malachite green in aquatic animals. J Chromatogr B Biomed Appl. 2003;788: 351–359. 10.1016/s1570-0232(03)00042-4 12705975

[2] Zhang C, Zhang S, Diao H, Zhao H, Zhu X, Lu F, et al Purification and characterization of a temperature- and pH-stable laccase from the spores of *Bacillus vallismortis* fmb-103 and its application in the degradation of malachite green. J Agric Food Chem. 2013;61: 5468–5473. 10.1021/jf4010498 23706133

[3] Srivastava S, Sinha R, Roy D. Toxicological effects of malachite green. Aquat Toxicol. 2004;66: 319–329. 15129773 10.1016/j.aquatox.2003.09.008

[4] Tamayo-Ramos JA, van Berkel WJ, de Graaff LH. Biocatalytic potential of laccase-like multicopper oxidases from *Aspergillus niger* . Microb Cell Fact. 2012;11: 165 10.1186/1475-2859-11-165 23270588 PMC3548707

[5] Cha C-J, Doerge DR, Cerniglia CE. Biotransformation of malachite green by the fungus *Cunninghamella elegans* . Appl Environ Microbiol. 2001;67: 4358–4360. 11526047 10.1128/AEM.67.9.4358-4360.2001 PMC93171

[6] Kalyani DC, Patil PS, Jadhav JP, Govindwar SP. Biodegradation of reactive textile dye Red BLI by an isolated bacterium *Pseudomonas* sp. SUK1. Bioresour Technol. 2008;99: 4635–4641. 17765541 10.1016/j.biortech.2007.06.058

[7] Rahman I, Saad B, Shaidan S, Sya Rizal E. Adsorption characteristics of malachite green on activated carbon derived from rice husks produced by chemical–thermal process. Bioresour Technol. 2005;96: 1578–1583. 15978990 10.1016/j.biortech.2004.12.015

[8] Berberidou C, Poulios I, Xekoukoulotakis N, Mantzavinos D. Sonolytic, photocatalytic and sonophotocatalytic degradation of malachite green in aqueous solutions. Appl Catal B. 2007;74: 63–72.

[9] Chen C, Lu C, Chung Y, Jan J. UV light induced photodegradation of malachite green on TiO_2_ nanoparticles. J Hazard Mater. 2007;141: 520–528. 16934397 10.1016/j.jhazmat.2006.07.011

[10] Forgacs E, Cserháti T, Oros G. Removal of synthetic dyes from wastewaters: a review. Environ Int. 2004;30: 953–971. 15196844 10.1016/j.envint.2004.02.001

[11] Azmi W, Sani RK, Banerjee UC. Biodegradation of triphenylmethane dyes. Enzyme Microb Technol. 1998;22: 185–191. 9463944 10.1016/s0141-0229(97)00159-2

[12] Jang M-S, Lee Y-M, Kim C-H, Lee J-H, Kang D-W, Kim S-J, et al Triphenylmethane reductase from *Citrobacter* sp. strain KCTC 18061P: purification, characterization, gene cloning, and overexpression of a functional protein in *Escherichia coli* . Appl Environ Microbiol. 2005;71: 7955–7960. 16332773 10.1128/AEM.71.12.7955-7960.2005 PMC1317337

[13] Jasińska A, Różalska S, Bernat P, Paraszkiewicz K, Długoński J. Malachite green decolorization by non-basidiomycete filamentous fungi of *Penicillium pinophilum* and *Myrothecium roridum* . Int Biodeterior Biodegrad. 2012;73: 33–40.

[14] Wang J, Gao F, Liu Z, Qiao M, Niu X, Zhang K-Q, et al Pathway and molecular mechanisms for malachite green biodegradation in *Exiguobacterium* sp. MG2. PLoS One. 2012;7: e51808 10.1371/journal.pone.0051808 23251629 PMC3522578

[15] Maalej-Kammoun M, Zouari-Mechichi H, Belbahri L, Woodward S, Mechichi T. Malachite green decolourization and detoxification by the laccase from a newly isolated strain of *Trametes* sp. Int Biodeterior Biodegrad. 2009;63: 600–606.

[16] Murugesan K, Yang I-H, Kim Y-M, Jeon J-R, Chang Y-S. Enhanced transformation of malachite green by laccase of *Ganoderma lucidum* in the presence of natural phenolic compounds. Appl Microbiol Biotechnol. 2009;82: 341–350. 10.1007/s00253-008-1819-1 19130052

[17] Benyounes S, Bouallagui Z, Gargoubi A, Sayadi S. Investigation of dyes degradation intermediates with *Scytalidium thermophilum* laccase. Eur Food Res Technol. 2011;233: 751–758.

[18] Chhabra M, Mishra S, Sreekrishnan TR. Laccase/mediator assisted degradation of triarylmethane dyes in a continuous membrane reactor. J Biotechnol. 2009;143: 69–78. 10.1016/j.jbiotec.2009.06.011 19539671

[19] Kumar VV, Sathyaselvabala V, Premkumar MP, Vidyadevi T, Sivanesan S. Biochemical characterization of three phase partitioned laccase and its application in decolorization and degradation of synthetic dyes. J Mol Catal B: Enzym. 2012;74: 63–72.

[20] Balan K, Sathishkumar P, Palvannan T. Decolorization of malachite green by laccase: Optimization by response surface methodology. J Taiwan Inst Chem Eng. 2012;43: 776–782.

[21] Kang HW, Yang YH, Kim SW, Kim S, Ro H-S. Decolorization of triphenylmethane dyes by wild mushrooms. Biotechnol Bioproc E. 2014;19: 519–525.

[22] Wong DW. Structure and action mechanism of ligninolytic enzymes. Appl Biochem Biotechnol. 2009;157: 174–209. 10.1007/s12010-008-8279-z 18581264

[23] Piontek K, Antorini M, Choinowski T. Crystal structure of a laccase from the fungus *Trametes versicolor* at 1.90-Å resolution containing a full complement of coppers. J Biol Chem. 2002;277: 37663–37669. 12163489 10.1074/jbc.M204571200

[24] Couto SR, Herrera JLT. Industrial and biotechnological applications of laccases: A review. Biotechnol Adv. 2006;24: 500–513. 16716556 10.1016/j.biotechadv.2006.04.003

[25] Papinutti VL, Forchiassin F. Modification of malachite green by *Fomes sclerodermeus* and reduction of toxicity to *Phanerochaete chrysosporium* . FEMS Microbiol Lett. 2004;231: 205–209. 14987766 10.1016/S0378-1097(03)00957-1

[26] Morozova OV, Shumakovich GP, Shleev SV, Yaropolov YI. Laccase-mediator systems and their applications: A review. Appl Biochem Microbiol. 2007;43: 523–535. 18038679

[27] Solomon EI, Chen P, Metz M, Lee S-K, Palmer AE. Oxygen binding, activation, and reduction to water by copper proteins. Angew Chem Int Ed. 2001;40: 4570–4590. 12404359 10.1002/1521-3773(20011217)40:24<4570::aid-anie4570>3.0.co;2-4

[28] Pogni R, Baratto MC, Sinicropi A, Basosi R. Spectroscopic and computational characterization of laccases and their substrate radical intermediates. Cell Mol Life Sci. 2015;72: 885–896. 10.1007/s00018-014-1825-7 25595303 PMC11113710

[29] Yang J, Lin Q, Ng TB, Ye X, Lin J. Purification and characterization of a novel laccase from *Cerrena* sp. HYB07 with dye decolorizing ability. PLoS One. 2014;9: e110834 10.1371/journal.pone.0110834 25356987 PMC4214704

[30] Daâssi D, Frikha F, Zouari-Mechichi H, Belbahri L, Woodward S, Mechichi T. Application of response surface methodology to optimize decolourization of dyes by the laccase-mediator system. J Environ Manage. 2012;108: 84–91. 10.1016/j.jenvman.2012.04.039 22659603

[31] Roriz MS, Osma JF, Teixeira JA, Rodríguez Couto S. Application of response surface methodological approach to optimise Reactive Black 5 decolouration by crude laccase from *Trametes pubescens* . J Hazard Mater. 2009;169: 691–696. 10.1016/j.jhazmat.2009.03.150 19409701

[32] Sathishkumar P, Balan K, Palvannan T, Kamala-Kannan S, Oh B-T, Rodríguez-Couto S. Efficiency of *Pleurotus florida* laccase on decolorization and detoxification of the reactive dye Remazol Brilliant Blue R (RBBR) under optimized conditions. Clean (Weinh). 2013;41: 665–672.

[33] Saravanakumar T, Palvannan T, Kim D-H, Park S-M. Manganese peroxidase h4 isozyme mediated degradation and detoxification of triarylmethane dye malachite green: optimization of decolorization by response surface methodology. Appl Biochem Biotechnol. 2013;171: 1178–1193. 10.1007/s12010-013-0220-4 23604969

[34] Fischer AR, Werner P, Goss K-U. Photodegradation of malachite green and malachite green carbinol under irradiation with different wavelength ranges. Chemosphere. 2011;82: 210–214. 10.1016/j.chemosphere.2010.10.019 21035831

[35] Eggert C, LaFayette PR, Temp U, Eriksson K-EL, Dean JFD. Molecular analysis of a laccase gene from the white rot fungus *Pycnoporus cinnabarinus* . Appl Environ Microbiol. 1998;64: 1766–1772. 9572949 10.1128/aem.64.5.1766-1772.1998 PMC106228

[36] Parshetti G, Kalme S, Saratale G, Govindwar S. Biodegradation of malachite green by *Kocuria rosea* MTCC 1532. Acta Chim Slov. 2006;53: 492–498.

[37] Du L-N, Wang S, Li G, Wang B, Jia X-M, Zhao Y-H, et al Biodegradation of malachite green by *Pseudomonas* sp. strain DY1 under aerobic condition: characteristics, degradation products, enzyme analysis and phytotoxicity. Ecotoxicology. 2011;20: 438–446. 10.1007/s10646-011-0595-3 21253837

